# Effects of the Combination of Protein in the Internal Aqueous Phase and Polyglycerol Polyricinoleate on the Stability of Water-In-Oil-In-Water Emulsions Co-Encapsulating Crocin and Quercetin

**DOI:** 10.3390/foods13010131

**Published:** 2023-12-29

**Authors:** Wei Fan, Yan Shi, Yueming Hu, Jing Zhang, Wei Liu

**Affiliations:** 1 State Key Laboratory of Food Science and Resources, Nanchang University, 235 Nanjing East Road, Nanchang 330047, China; fanwei1726151911@163.com (W.F.); huyueming@ncu.edu.cn (Y.H.); 417900220112@email.ncu.edu.cn (J.Z.); 17351028241@163.com (W.L.); 2 Department of Food Science and Engineering, Nanchang University, 235 Nanjing East Road, Nanchang 330047, China

**Keywords:** W/O/W emulsion, plant protein, synergistic effect, encapsulation properties, bioavailability

## Abstract

This study aimed to diminish the reliance on water-in-oil-in-water (W/O/W) emulsions on the synthetic emulsifier polyglycerol polyricinoleate (PGPR). Considering the potential synergistic effects of proteins and PGPR, various protein types (whey, pea and chickpea protein isolates) were incorporated into the internal aqueous phase to formulate W/O/W emulsions. The effects of the combination of PGPR and protein at different ratios (5:0, 4:1, 3:2, 1:1 and 2:3) on the stability and encapsulation properties of W/O/W emulsions co-encapsulating crocin and quercetin were investigated. The findings indicated that the combination of PGPR and protein resulted in a slight reduction in the encapsulation efficiency of the emulsions, compared to that of PGPR (the control). Nonetheless, this combination significantly enhanced the physical stability of the emulsions. This result was primarily attributed to the smaller droplet sizes and elevated viscosity. These factors contributed to increased retentions of crocin (exceeding 70.04%) and quercetin (exceeding 80.29%) within the emulsions after 28 days of storage, as well as their improved bioavailability (increases of approximately 11.62~20.53% and 3.58~7.98%, respectively) during gastrointestinal digestion. Overall, combining PGPR and protein represented a viable and promising strategy for reducing the amount of PGPR and enhancing the stability of W/O/W emulsions. Notably, two plant proteins exhibited remarkable favorability in this regard. This work enriched the formulations of W/O/W emulsions and their application in the encapsulation of bioactive substances.

## 1. Introduction

Recently, there has been a focus on the co-encapsulation of multiple bioactive substances within single food matrices, due to the synergistic effect of bioactivities. However, co-encapsulating bioactive compounds with varying solubilities within the same system is still a challenge, particularly when dealing with hydrophilic and hydrophobic substances. It is well known that water-in-oil-in-water (W/O/W) emulsions, characterized by a distinctive two-membrane and three-phase structure, offer an innovative and promising encapsulation approach for bioactive substances in the food industry [1]. This emulsion type effectively co-encapsulates both hydrophilic and hydrophobic substances, ensuring efficient protection and controlled release.

However, the thermodynamic instability of W/O/W emulsions presents a significant obstacle to their commercial application in the food industry. The movement of droplets within the internal and external aqueous phases results in emulsion instability [2]. To overcome such problems, the addition of emulsifiers with a low-level hydrophile–lipophile balance can reduce the interfacial energy at the oil–water interface and stabilize emulsions [3]. Currently, the most widely used oil-soluble emulsifier in the food industry is polyglycerol polyricinoleate (PGPR) [4]. However, PGPR, a synthetic emulsifier, has legal daily intake restrictions and its taste limits its acceptance among consumers [5]. To satisfy consumers’ demands for healthy and natural products, natural ingredients are increasingly encouraged to replace PGPR in food products.

There are two popular current strategies to remove PGPR in W/O/W emulsions. On the one hand, another hydrophobic emulsifier can replace PGPR directly or in a compound with PGPR, such as ammonium phosphatide [6], sucrose esters [7] and lecithin [8]. However, the W/O/W emulsions formulated with these emulsifiers are less stable than those of PGPR. Additionally, the selection of natural emulsifiers is limited. On the other hand, the combination of proteins or polysaccharides with PGPR is employed. It has been suggested that there may be a synergistic enhancing effect between PGPR and protein, favoring stability, such as sodium caseinate [9], β-lactoglobulin [10] and a pectin–pea protein isolate conjugate [11]. Therefore, employing natural constituent proteins to replace PGPR, rather than relying on other lipophilic emulsifiers, represents a favorable alternative. Nonetheless, the utilization of proteins in this regard remains limited. Specifically, no report exists regarding the incorporation of plant proteins into the internal aqueous phase of W/O/W emulsions to replace PGPR. In recent years, plant proteins have emerged as a sustainable alternative to animal proteins [12]. Legume ranks as the world’s second most popular crop and serves as an abundant source of plant protein [13]. Legume proteins exhibit versatility, providing nutrition and sustainability, thereby serving as apt protein sources to craft functional ingredient emulsions and microencapsulation systems [14,15]. Soybean isolate proteins, which contain abundant nutrients and boast exceptional functional properties, are extensively utilized as food ingredients and additives in the food industry [16]. Nevertheless, in response to the growing call for sustainable plant proteins, pea (PPI) and chickpea protein isolations (CPI) are gaining preference, due to their acknowledged biocompatibility, nutritional value, exceptional functionality and hypoallergenic nature [17,18].

The development of functional food emulsions has become a popular research topic, due to the vulnerability of functional ingredients and the unique protective structure of W/O/W emulsions [19]. Crocin is a natural hydrophilic carotenoid that possesses a variety of pharmacological properties, including anti-obesity, -depression and -cancer properties [20]. Nevertheless, crocin exhibits susceptibility to adverse environments (related to light, temperature, acidic pH and oxygen) during processing and storage, leading to its degradation [21]. Quercetin is an excellent natural anti-oxidant with a range of physiological activities, such as anti-cancer, viral and inflammatory properties [22]. However, the low-level water solubility, gastrointestinal absorption and bioavailability of quercetin pose challenges for its application in food matrices [23]. A previous study demonstrated the synergistic effect of combining crocin and quercetin in combating obesity [24]. Therefore, employing a W/O/W emulsion to co-encapsulate crocin and quercetin represents an essential means to achieve the synergistic enhancement of bioactivity, stability and release characteristics.

In this study, PPI and CPI were introduced in the internal aqueous phase and used in proportion with PGPR in the oil phase, preparing W/O/W emulsions co-encapsulating crocin and quercetin. Whey protein isolate (WPI), a commonly employed animal protein emulsifier, was used in comparison with PPI and CPI. The primary objective was to explore the potential synergistic effects between the proteins and PGPR, while diminishing our reliance on PGPR. The physical stability, encapsulation and interfacial characterization of W/O/W emulsions, stabilized by a combination of proteins and PGPR, were investigated. Furthermore, the effects of the combination of PGPR and proteins on the storage stability and bioavailability of the encapsulated substances in W/O/W emulsions were evaluated.

## 2. Materials and Methods

### 2.1. Materials

Whey (WPI, 80%) and pea protein isolates (PPI, 80%) were acquired from Shanghai Yuanye Biotechnology Co., Ltd., (Shanghai, China). Chickpea protein isolate (CPI, 80%) was purchased from Shaanxi Jintaikang Biotechnology Co., Ltd., (Xian, China). Crocin (>98%), quercetin (>98%), olive oil, polyglycerol polyricinoleate (PGPR) and pectin were acquired from Shanghai McLean Biochemical Technology Co., Ltd., (Shanghai, China). The other reagents were of analytical grade.

### 2.2. Preparation of Water-In-Oil-In-Water Emulsions Co-Encapsulating Crocin and Quercetin

#### 2.2.1. Preparation of Solutions

Crocin (0.2%, *w*/*v*), NaCl (2%, *w*/*v*) and proteins were dissolved in deionized water (pH 7.0) to obtain the internal aqueous phase (W_1_). PGPR was added to olive oil at 60 °C under magnetic stirring for 30 min until it was fully dissolved. Then, an ethanol solution of 5 mg/mL quercetin was added dropwise to olive oil and stirred at 65 °C until all the ethanol evaporated, to obtain the oil phase (O) containing 1 mg/mL quercetin. The ratios of PGPR and proteins were 5:0, 4:1, 3:2, 1:1 and 2:3, respectively. The external aqueous phase (W_2_) was prepared by mixing the WPI and pectin (PEC) solutions at a ratio of 3:1, adjusting the pH of the mixed solution to 3.5 with HCl [25]. The mixed solution was stirred for 30 min at room temperature and overnight at 4 °C to obtain the WPI–PEC complex solution (WPI 3%, PEC 1%, *w*/*v*).

#### 2.2.2. Preparation of Water-In-Oil-In-Water Emulsions

A two-step emulsification method was used to prepare W/O/W emulsions. First, W_1_ (30%, *w*/*w*) was slowly added to O (70%, *w*/*w*) with a T18 digital Ultra-Turrax (IKA, Staufen, Germany) at 6000 rpm. The mixture was subjected to shear dispersion for 3 min at 20,000 rpm, followed by high-pressure homogenization at 30 MPa for 2 min. The W/O emulsion was finally obtained. The W/O emulsion (40%, *w*/*w*) obtained in the first step was added to W_2_ (60%, *w*/*w*). The mixture was homogenized using the T18 digital Ultra-Turrax at 10,000 rpm for 4 min to formulate the W/O/W emulsion. Sodium azide (0.01%, *w*/*v*) was introduced into the final W/O/W emulsion. In the prepared emulsions, P/WPI, P/PPI and P/CPI represented the combination of PGPR and proteins (WPI, PPI and CPI), respectively.

### 2.3. Effects of Polyglycerol Polyricinoleate Concentration on the Characteristics of Water-In-Oil-In-Water Emulsions

Different concentrations of PGPR (0%, 1%, 3%, 5%, 7% and 9%, *w*/*w*) were dissolved in olive oil to obtain the W/O/W emulsions. To determine the optimal PGPR concentration for the preparation of W/O/W emulsions, the physical stability and particle size of the emulsions were determined and characterized with a multiple light scattering instrument and Malvern laser particle sizer, respectively. The selected optimal concentration of PGPR was used as the total concentration of PGPR and protein in subsequent experiments. This optimal concentration was considered as the control.

### 2.4. Characterization of Water-In-Oil-In-Water Emulsions

#### 2.4.1. Determination of Particle Size and Zeta Potential (ζ-Potential)

The volume-weighted mean diameter (D [4,3]) was determined using a Malvern laser particle sizer (Mastersizer 3000, Malvern Instrument Co., Ltd., Worcestershire, UK). The refractive indices of droplets and deionized water were set to 1.470 and 1.330. The ζ-potential of the emulsions was determined using a Zetasizer nano (Malvern Zetasizer Nano-ZS, Malvern Instruments Co., Ltd., Worcestershire, UK). The samples were diluted 400 times with deionized water.

#### 2.4.2. Microstructure

The W/O/W emulsions were diluted with deionized water at a 1:100 ratio and subsequently examined with a 40× objective lens under the optical microscope (Eclipse Ni-U, Nikon Co., Ltd., Tokyo, Japan).

The microstructure of the W/O/W emulsion was examined using Confocal laser scanning microscopy (CLSM) (TCS SP8, Leica Microsystems GmbH, Wetzlar, Germany) [26]. The proteins were stained with Nile blue (0.01%, *w/v*, dissolved in isopropanol), while the oils were stained with Nile red (0.01%, *w/v*, dissolved in isopropanol). A mixed dye solution (1:1, *v/v*) of approximately 100 μL was added to 1 mL of the sample and left to stain for 20 min in the dark. The stained sample (10 μL) was applied to a slide and observed by CLSM under a 30× objective lens. Nile blue was excited at 633 nm, while Nile red was excited at 488 nm.

#### 2.4.3. Physical Stability

The physical stability of W/O/W emulsions was characterized using a multiple light scattering instrument (TURBISCAN Lab, Formulaction, Toulouse, France). The instrument performed a static vertical scan of the sample through two detectors (transmission and backscattering light) and monitored the flocculation and upwelling of the emulsion. Each emulsion (30 mL) was added to the test tubes and the scanning lasted for 24 h at 25 °C. The scanning interval was 1 h. Backscattering light was exclusively used for analysis, as the W/O/W emulsion’s opacity prevents the transmission of light. The formulas for BS and TSI are as follows:

$$\mathrm{BS}=\frac{1}{\sqrt{\lambda^{*}}}$$

$$\lambda^{*}(\phi ,\;d)=\frac{2d}{3\varphi \left(1-g\right)Q_{s}}$$

where λ* represents the free path of the photon transmission, d represents the average diameter of the particle, φ represents the volume concentration of the particle and g and Q_S_ represent the optical parameters of Mie theory.

The Turbiscan stability index (TSI) was employed to assess the physical stability of emulsions and was calculated as follows:

$$\mathrm{TSI}=\sum_{n}\frac{\sum_{h}|{\mathrm{scan}}_{n}\left(h\right)\;-\;{\mathrm{scan}}_{n-1}\left(h)\right|}{H}$$

where scan_n_ (h) and scan_n−1_ (h) represent the intensity of scanning light, n represents the setting scan time, h represents the height of the test tubes and H represents the sample height.

#### 2.4.4. Encapsulation Efficiency

The encapsulation efficiency of crocin and quercetin was determined using Hu et al.’s method [27], with several adaptations. The W/O/W emulsions were mixed with a water solution containing 25% (*v/v*) ethanol and 0.5% (*v/v*) Tween 80. The mixture was then stirred until it dissolved, followed by centrifugation at 13,000 rpm for 10 min (4 °C) (Anting Scientific Instrument, Shanghai, China). The solution at the bottom was collected, and then subjected to centrifugation under the same conditions. The obtained solution at the bottom was filtered using a 0.45 μm filter. The absorbance of crocin was measured at 440 nm and the quercetin was measured at 373 nm, using double-beam Ultraviolet (UV)-vis spectroscopy (U-2910, Hitachi Co., Ltd., Tokyo, Japan). The standard curves of crocin and quercetin were established, to calculate the crocin and quercetin content. The encapsulation efficiency (EE) was calculated as follows:

(1) $$\mathrm{EE}\left(\%\right)=(1-\frac{M}{M_{0}})\times 100\%$$

where M represents the amount of crocin or quercetin within the obtained solution at the bottom (mg). M_0_ represents the amount of crocin or quercetin added to the emulsion (mg).

### 2.5. General Properties of Proteins

#### 2.5.1. Particle Size and ζ–Potential

The measurements of protein particle size and ζ–potential were conducted using a Malvern particle sizer and Zetasizer nano, respectively.

#### 2.5.2. Surface Hydrophobicity

The sample solution was diluted to a solution with a certain concentration gradient (0.25, 0.5, 1.0, 2.0 and 4.0 mg/mL). The diluted sample solution (4 mL) was mixed with 50.0 μL of 1,8-anilinonaphthalenesulfonate (ANS) solution (8 mM, pH 7.0) and reacted for 15 min away from light. The fluorescence intensity of each sample was tested at the excitation wavelength of 390 nm and the emission wavelength of 470 nm, using a fluorescence spectrophotometer (F-7000, Hitachi Co., Tokyo, Japan). The slit and scanning speed were 5 nm and 1200 nm/min, respectively. The protein concentration and fluorescence intensity were plotted as horizontal and vertical coordinates, respectively. The initial slope was determined through linear regression analysis, which served as an indicator of surface hydrophobicity.

### 2.6. Interfacial Properties of Water-In-Oil-In-Water Emulsions

#### 2.6.1. Rheological Properties

The rheological properties of emulsions were assessed using a dynamic shear rheometer (MCR302, Anton Paar, Graz, Austria) according to Huang et al.’s protocol [25]. Each sample was placed between two parallel plates. The examination of viscosity was conducted across a range of shear rates from 0.1 to 100 s^−1^ at 25 °C. The storage (G′) and loss (G″) moduli were obtained through dynamic oscillatory measurements at an oscillation frequency from 0.1 to 10 Hz at 25 °C and with 1% strain.

#### 2.6.2. Interfacial Tension

The interfacial tension between the internal water aqueous and the oil aqueous was determined using OCA25 (Dataphysics Instruments Co., Ltd., Filderstadt, Germany). The internal water and oil phases were added to the syringe and quartz dish, respectively. The samples were equilibrated for 30 min before testing. A water droplet of 15 μL was formed at the tip of the injection capillary. The droplet profile was continuously monitored for 60 min using a video camera at 25 °C while the interfacial tension was calculated.

### 2.7. Storage Stability of Water-In-Oil-In-Water Emulsions Co-Encapsulating Crocin and Quercetin

The equal concentrations of crocin aqueous and quercetin ethanol solutions, and the freshly prepared W/O/W emulsions of co-encapsulated crocin and quercetin, were stored for 28 days at 25 °C, protected from light. The retention rate was determined every 7 days. The retention rate of crocin and quercetin was quantified using Tian et al.’s method [28]. The W/O/W emulsion (1 mL) was mixed with 4 mL of absolute ethanol and then sonicated at 200 W for 10 min to completely demulsify the emulsion. The mixture was centrifuged at 8000 rpm for 10 min (4 °C). The obtained supernatant was filtered through a 0.45 μm filter. The method used to determine the absorbance and calculation is the same as in Section 2.4.4. The retention rate (RE) of W/O/W emulsions was calculated as follows:

(2) $$\mathrm{RE}\left(\%\right)=\frac{C}{C_{0}}\times 100\%$$

where C and C_0_ represent the content of crocin or quercetin in freshly made W/O/W emulsions and during storage, respectively.

### 2.8. In Vitro Digestion

In vitro simulated digestion experiments were conducted following the methodology outlined by Huang et al. [29], with some adaptations. Simulated gastric fluid (SGF) (3.2 mg/mL pepsin, 2.0 mg/mL NaCl and 0.7% (*v/v*) HCl, pH 1.2) was first prepared. A total of 20 mL of the sample was mixed with 20 mL of SGF and the pH was adjusted to 2.0 with 1 M NaOH. The temperature was kept constant at 37 °C, under magnetic stirring at 100 r/min, for 2 h. The pH of the mixed system was quickly adjusted to 7.0 with 0.25 M NaOH, to deactivate the pepsin. The gastric fluid was homogenously mixed with simulated intestinal fluid (SIF, containing 10 mM CaCl_2_, 150 mM NaCl, 5 mg/mL porcine bile salt, 1.6 mg/mL porcine pancreatic and 3.2 mg/mL lipase, pH 7.0), and the pH of the mixture was adjusted to 7.0 with 0.25 M NaOH. The temperature was kept constant at 37 °C and then the mixture was magnetically stirred at 100 r/min for 2 h.

The oil phase underwent hydrolysis in the small intestine, leading to the formation of free fatty acids (FFA), which were then tracked by measuring the amount of NaOH usage. The percentage of FFA releases was calculated as follows:

(3) $$\mathrm{FFA}\left(\%\right)=\frac{V_{\mathrm{NaOH}}\times C_{\mathrm{NaOH}}\times M_{\mathrm{Lipid}}}{W_{\mathrm{Lipid}}\times 2}\times 100\%$$

where V_NaOH_ represents the amount of NaOH usage (L), C_NaOH_ represents the concentration of NaOH (mol/L), M_Lipid_ represents the mass of the olive oil (g) and W_Lipid_ represents the molar mass of the olive oil (g/mol).

The digested mixture was centrifuged at 12,000 rpm for 20 min (4 °C). The obtained micellar layer was filtered through a 0.45 μm filter. The method for determination of absorbance and calculation of amount is the same as in Section 2.4.4. Bioavailability was calculated as follows:

(4) $$\mathrm{Bioavailability}\;\left(\%\right)=\frac{C_{M}}{C_{0}}\times 100\%$$

where C_M_ and C_0_ represent the amounts of crocin or quercetin in the micelle layer and the emulsion, respectively.

### 2.9. Statistical Analysis

The experimental results are reported as the mean ± standard deviation. The data were analyzed through Duncan tests, using SPSS 26.0 statistical software (with a significance level of *p* < 0.05). All measurements above were performed in triplicate.

## 3. Results and Discussion

### 3.1. Effects of Polyglycerol Polyricinoleate Concentration on the Stability of Water-In-Oil-In-Water Emulsions

**Figure 1:**
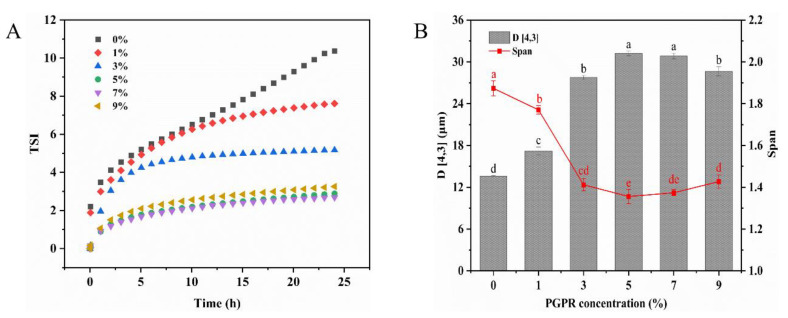
(**A**) Turbiscan stability index value (TSI) curves and (**B**) mean droplet diameter (D [4,3]) of W/O/W emulsions stabilized by different concentrations of PGPR. The values are shown as means ± SD of triplicate determinations. Different lowercase letters represent significant differences (*p* < 0.05) between the different W/O/W emulsions with different PGPR concentrations.

The Turbiscan stability index (TSI) value can be used to evaluate the global stability of the emulsion. Generally, a more stable emulsion system is indicated by a lower TSI and a slower rate of change [30]. The results indicated that the TSI values decreased with an increasing PGPR concentration (Figure 1A). Compared to 1% (*w/w*) PGPR, the TSI value decreased significantly when the PGPR concentration was 5% (*w/w*). This decline can likely be attributed to the substantial adsorption of PGPR at the water–oil interface, which ensured ample emulsifier coverage for the internal water droplets, thereby constraining their release and consequently enhancing the stability of the W/O/W emulsions. When further elevating the PGPR concentration to 7% (*w/w*), there was no significant alteration in the TSI values. Additionally, the stability of the W/O/W emulsions is directly correlated with the particle size. As demonstrated in Figure 1B, the droplets in the W/O/W emulsions became significantly larger as the PGPR concentration increased. When the PGPR concentration increased to 5% and 7% (*w/w*), no significant changes in droplet size were observed (*p* > 0.05). However, a further increase to 9% (*w/w*) in the PGPR concentration led to a reduction in the particle size. This phenomenon may be attributed to excess PGPR, potentially triggering the spontaneous formation of reverse micelles and the subsequent release of internal water droplets in the external water phase [31]. This provides an explanation for the increase in TSI values in the emulsions containing 9% (*w/w*) PGPR. The span index of particle sizes in the emulsion first decreased and then increased with PGPR concentration. At a PGPR concentration of 5% (*w/w*), the span index was minimal. This suggested a more uniform particle distribution within the emulsion at this point. Therefore, the W/O/W emulsion prepared with a 5% (*w/w*) PGPR concentration exhibited a uniform particle size distribution and demonstrated excellent physical stability. The 5% PGPR concentration was used as the total concentration of PGPR and protein in the subsequent experiments, while this concentration was also considered as the control.

### 3.2. Optical Microscopy and Encapsulation Efficiency of Water-In-Oil-In-Water Emulsions Stabilized by the Combination of Proteins and Polyglycerol Polyricinoleate

**Figure 2:**
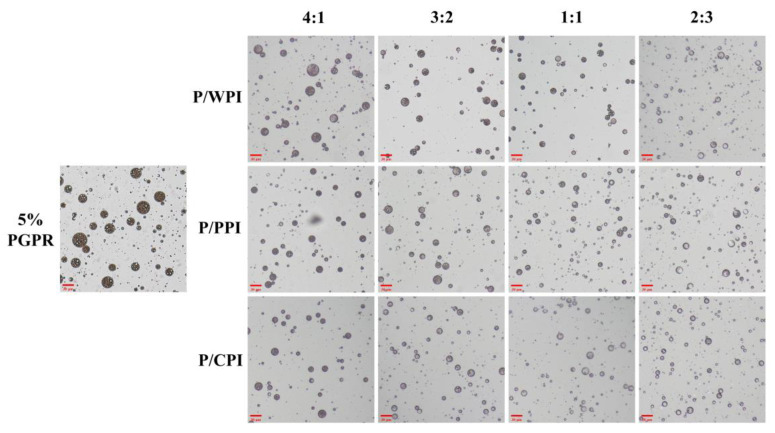
Optical microscope images of W/O/W emulsions at different PGPR/protein ratios. P/WPI, P/PPI and P/CPI represent the combination of PGPR with WPI, PPI and CPI, respectively.

Optical microscope images of the W/O/W emulsions with PGPR and proteins at different ratios are presented in Figure 2. A substantial reduction was observed in droplet size upon the incorporation of PGPR with the protein. When the ratios of PGPR/protein were 4:1 and 3:2, the oil droplets were darker in color and appeared as black areas, implying that they were filled with water droplets. The areas were black due to the small inner water droplets below one micron being out of focus levels [32]. However, as the percentage of proteins increased, the oil droplets became lighter, and the sizes of the W/O/W emulsion droplets decreased. This suggests that the water droplets were not fully encapsulated. A notable portion of water droplets remained unencapsulated by the oil droplets at the ratios of 1:1 and 2:3, consequently preventing the formation of W/O/W emulsions. The results reflect variations in the encapsulation efficiency of crocin.

As shown in Table 1, the encapsulation efficiency of crocin in a W/O/W emulsion containing 5% (*w/w*) PGPR (control) reached 86.90%. With the addition of the protein, the encapsulation efficiency of crocin decreased. For the emulsions co-stabilized with proteins and PGPR, the order of encapsulation efficiencies was WPI > PPI > CPI. Notably, at a PGPR/protein ratio of 4:1, the encapsulation efficiency was higher than the emulsion with 4% PGPR (77.76%) (Table S1). Comparable results were observed at a ratio of 3:2. However, when the ratios were 1:1 and 2:3, the encapsulation rate was lower than those of the emulsions stabilized with 2.5% and 2% PGPR. The combination of PGPR and protein decreased quercetin’s encapsulation efficiency in the emulsion by approximately 2.09%, to 6.48%, which is slightly below the control (94.89%), exhibiting a decrease ranging from 2.09% to 6.48%. Interestingly, the encapsulation efficiency of quercetin was unaffected by the type of protein used (Table 1). In brief, the reduction in the encapsulation rate of crocin and quercetin in the emulsions was less pronounced when the PGPR content was reduced by 1% and 2%. To further examine the impact of using both PGPR and proteins on the emulsion stability, while maintaining a higher encapsulation efficiency, the PGPR/protein ratios of 4:1 and 3:2 were selected.

### 3.3. Physical Stability of Water-In-Oil-In-Water Emulsions

**Figure 3:**
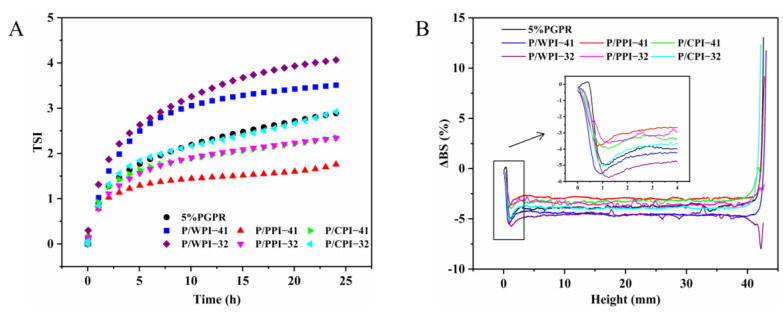
(**A**) TSI value curves and (**B**) delta backscattering (ΔBS) of W/O/W emulsions at PGPR/protein ratios of 4:1 and 3:2.

Figure 3 shows the impact of different proteins on the physical stability of W/O/W emulsions. As shown in Figure 3A, the TSI value of the W/O/W emulsion with pure PGPR was 2.89 after 24 h. When the ratio of PGPR to protein was 4:1, the TSI values of emulsions with PPI and CPI decreased to 1.76 and 2.35, respectively. The increase in the TSI value of the emulsions corresponded to an increase in the proportion of protein. However, the TSI values of PPI (2.34) remained lower than those of the control (2.89), and CPI (2.92) exhibited a minor elevation in comparison to the control at the ratio of 3:2. Based on these results, the combination of PPI or CPI and PGPR improved the physical stability of W/O/W emulsions. This is attributed to the proteins within the aqueous phase that mix with PGPR at the oil–water interface, augmenting the viscoelasticity and deformation resistance of the interfacial layer, which contributes significantly to the stability of the W/O/W emulsions [33]. Nevertheless, the TSI values for the emulsion containing WPI were higher than those of the control, suggesting that the combined use of PGPR and WPI negatively impacted the physical stability of the emulsion.

To further analyze the impacts of the combination of PGPR and proteins on the stability of emulsions, the ΔBS data of each sample are shown in Figure 3B. Some potential information about the emulsions can be derived from ΔBS, including instability trends and particle sizes. Clarification can be seen in the variation observed on the left side of the graph (bottom of the sample), while creaminess manifests in the variation on the top right side of the graph (top of the sample). In all the W/O/W emulsions, the ΔBS value exhibits a decrease at the bottom of the sample, denoted by a negative peak. This decrease signifies stratification at the emulsion’s bottom, accompanied by a reduction in particle concentration, resulting in emulsion clarification and the ΔBS value at the top of the sample increasing, indicating an elevated concentration of particles at the top, forming a creamy layer [34,35]. At the 4:1 and 3:2 ratios, the absolute values of ΔBS in emulsions with PPI or CPI were below those of the control after 24 h. This may be due to the increase in emulsion viscosity and decrease in droplet migration rate caused by the presence of PPI or CPI. A decrease or increase in the ΔBS value in the middle of the emulsion can indicate a change in particle size [36]. The W/O/W emulsions with PPI or CPI had an increase in the ΔBS value in the middle of the emulsion after 24 h compared to the control, implying their smaller particle sizes.

Overall, the combination of PGPR and PPI or CPI at PGPR/protein ratios of 4:1 and 3:2 favored the physical stability of the W/O/W emulsions. To comprehensively assess the stability of the emulsions, the analysis of several key physicochemical properties was conducted, encompassing particle size, ζ-potential, surface hydrophobicity, rheological properties and interfacial tension.

### 3.4. Droplet Size, ζ-Potential and Microstructure of Water-In-Oil-In-Water Emulsions

**Figure 4:**
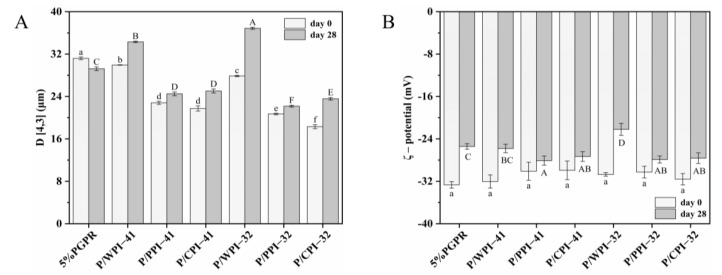
(**A**) Mean droplet diameter (D [4,3]) and (**B**) ζ–potential of W/O/W emulsions on day 0 and after 28 days of storage. The values are shown as means ± SD of triplicate determinations. Different lowercase letters represent significant differences (*p* < 0.05) between the different W/O/W emulsions on day 0. Different uppercase letters represent significant differences (*p* < 0.05) between different W/O/W emulsions on day 28.

The stability of the emulsion is significantly influenced by the droplet size. The particle size of emulsions both fresh and after 28 days of storage is shown in Figure 4A. The emulsions with proteins experienced a decrease (approximately 1.0~14.0 μm) in particle size on day 0. The added proteins could self-assemble at the interface, forming viscoelastic two-dimensional nanolayers [37]. This self-assembly process reduced the interfacial tension and contributed to the generation of smaller droplets. Furthermore, the interaction between bioactive substances and proteins may influence the particle size, which promotes protein unfolding and rapid absorption at the interface, forming smaller droplets [38].

For both PGPR/protein ratios, the emulsions exhibited the following particle size sequence, CPI < PPI < WPI, confirming the ΔBS analysis results. The droplet size of emulsions with different proteins may correlate with both their size and structural characteristics. The order of the particle size of emulsions is consistent with the order of the three proteins (Table 2). Zhang et al. [39] suggested that proteins with smaller particle sizes exhibit enhanced interfacial properties by diffusing more rapidly across the interface, ultimately leading to the formation of smaller emulsion droplets. As shown in Figure 4A, the particle sizes of all the emulsions except the control increased over 28 days. When combining PGPR and PPI, a relatively minor increase was observed in the particle sizes of emulsions, with an approximate range of 1.0~3.0 μm. This observation suggested an enhanced emulsion stability with reduced flocculation or coalescence, as supported in the CLSM images. The emulsions with WPI showed various destabilizing phenomena, including enlarged internal water droplets, the leakage of internal water droplets and the coalescence of oil droplets.

As shown in Figure 4B, no significant differences in ζ-potential were observed among the freshly prepared emulsions, which can be ascribed to the charge measured on the surface of the emulsion oil droplets, exclusively provided by the WPI–PEC complex. The absolute potential of all the emulsions decreased after 28 days of storage. The emulsions containing PPI or CPI exhibited a smaller decrease in absolute potential compared to those of the control and WPI, contributing to preserving the physical stability of these emulsions. The ζ-potential of the proteins significantly impacts the physical stability of the emulsions. The proteins adsorbed at the interface have the capability to create an electrostatic barrier, consequently enhancing emulsion stability via electrostatic repulsion [40].

### 3.5. Rheological Properties of Water-In-Oil-In-Water Emulsions

**Figure 5:**
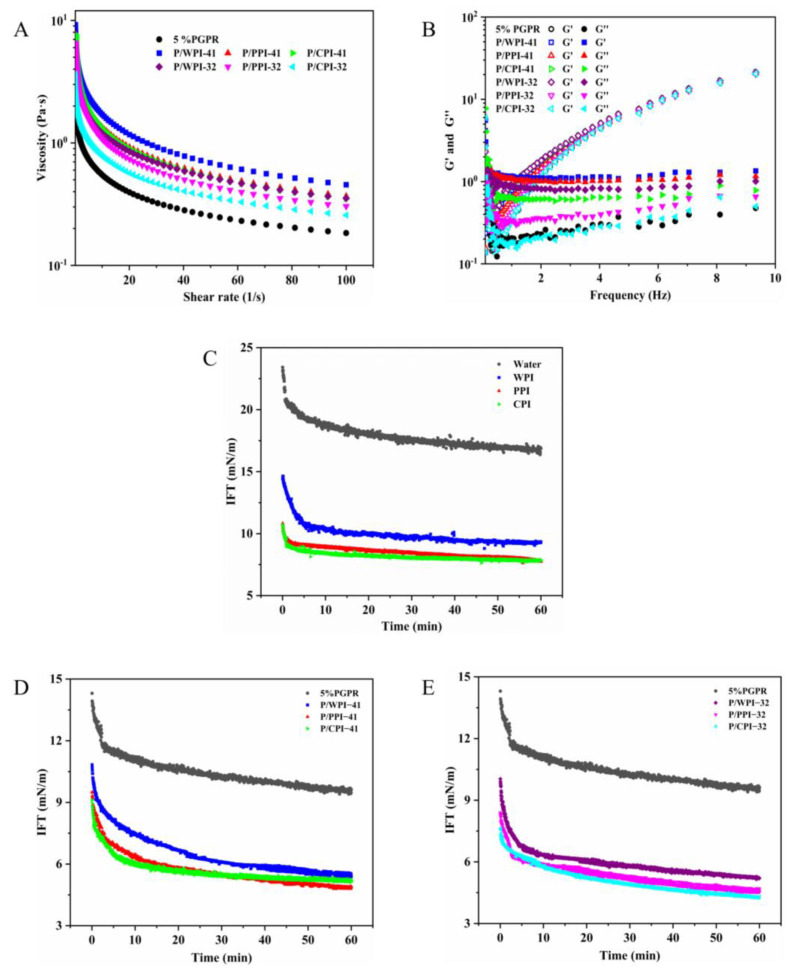
(**A**) Apparent viscosity, (**B**) dynamic storage modulus (G′) and loss modulus (G″) of W/O/W emulsions. The interfacial tension between the water and oil phase without PGPR (**C**) over 60 min. The interfacial tension between the water with proteins and oil phase with PGPR at PGPR/protein ratios of 4:1 (**D**) and 3:2 (**E**) over 60 min.

Rheological properties play a crucial role in assessing the texture and stability of emulsions. As depicted in Figure 5A, the viscosity of all the emulsions exhibited a decline as the shear rate increased. The non-Newtonian index (n) for the emulsions was less than one, which represents a pseudo-plastic fluid (Table S2) and revealed a shear-thinning behavior. This is because of the weak interactions among the droplets in the W/O/W emulsions. Consequently, as the shear rate increased, significant elongation, network fracture and droplet deformation took place. The k value represents the consistency factor of the emulsion; the larger the value of k is, the greater the viscosity of the emulsion is. According to the k value (Table S2), the inclusion of proteins in the internal water phase led to a more substantial increase in the viscosity of emulsions compared to that of the control (1.346), which is similar to the results reported by Tabibiazar and Hamishehkar [41]. The higher viscosity of emulsions with WPI (k-values of 4.229 and 3.175, respectively) at both ratios can be ascribed to the larger droplet size and the susceptibility to flocculation. In the flocculation process, the continuous phase particles became trapped, resulting in an increase in emulsion viscosity [42]. Simultaneously, flocculation facilitated droplet settling, which diminished the stability of the emulsions. As shown by the k value, there is no significant difference in the viscosity of the emulsions between PPI (3.589) and CPI (3.545) at a ratio of 4:1. The viscosity of the emulsions decreased as the percentage of protein went from 1% to 2%. These results also explain the changes in the TSI values.

Figure 5B depicts the storage (G′) and loss (G″) moduli of the W/O/W emulsions in the frequency sweep tests. G″ surpassed G′ at lower frequencies for all the emulsions, indicating a viscous fluid-like behavior. As the frequency increased, G′ and G″ gradually moved closer and intersected at a specific frequency point. At higher frequencies, G′ was larger than G″, demonstrating an elastic behavior that suggests the presence of a weak gel-like network in the emulsions [43]. This phenomenon is the benefit of the WPI–pectin complex in the external aqueous phase. The G′ and G″ of the emulsion increased slightly when the protein was present along with PGPR. This behavior might be ascribed to the reduction in droplet size within the emulsion, resulting in enhanced intermolecular stacking and less mobility [44]. These alterations contributed to heightened resistance to deformation, ultimately leading to the observed elevation in G′ and G″.

### 3.6. Interface Intension

The dynamic interfacial tension (IFT) reflects the ease with which an interfacial layer can form between the oil and water phases. As shown in Figure 5C, the interfacial tension between water and oil without PGPR reaches 16.87 mN/m over 60 min. The presence of proteins led to a reduction in the interfacial tension, with equilibrium interfacial tensions measured for WPI, PPI and CPI at 9.32, 7.79 and 7.82 mN/m, respectively. The addition of 5% PGPR resulted in a decrease in the interfacial tension between water and oil to 9.63 mN/m (Figure 5D), which was higher than that of the three proteins. However, the interfacial tension was further reduced in the emulsion system with proteins and PGPR. This reduction can be ascribed to the synergistic adsorption of the proteins and PGPR at the water–oil interface [45]. The interfacial tensions of WPI, PPI and CPI were reduced to 5.38, 4.53 and 4.31 mN/m at the PGPR/protein ratio of 3:2, respectively (Figure 5E). Protein molecules typically undergo stretching and conformational changes during adsorption. The rate and extent of stretching primarily rely on the flexibility of protein molecules [46]. Generally, high protein flexibility promotes a decrease in the interfacial tension. These mechanisms can account for the lower interfacial tension of PPI and CPI at the oil–water interface, compared to that of WPI. Furthermore, the interfacial tension could be related to surface hydrophobicity. The arrangement of hydrophobic amino acid residues on the protein molecule’s surface is the primary determinant of its surface properties. WPI exhibited the lowest surface hydrophobicity (Table 2), suggesting the limited presence of surface hydrophobic regions composed of hydrophobic residues. This hampered its adsorption at the oil–water interface. The above findings indicate a possible synergistic effect between the two components in the emulsion systems with the proteins and PGPR, leading to a noteworthy reduction in interfacial tension at the oil–water interface.

### 3.7. Storage Stability of Water-In-Oil-In-Water Emulsions Co-Encapsulating Crocin and Quercetin

**Figure 6:**
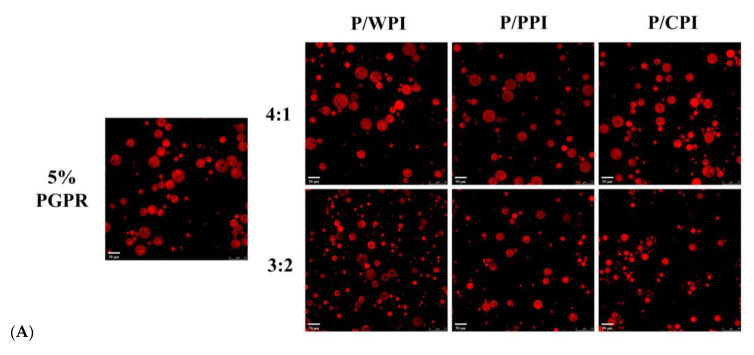

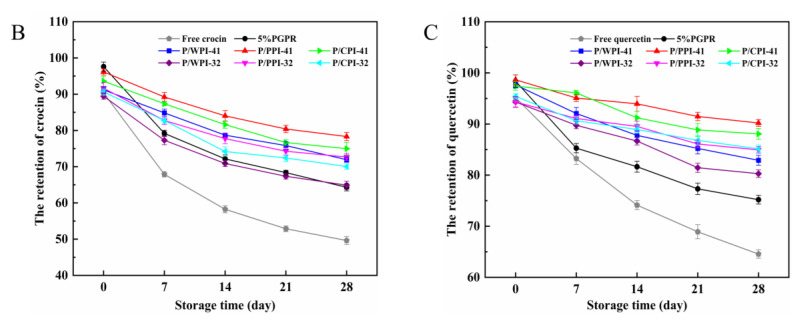
Confocal laser scanning microscopy images (**A**) of W/O/W emulsions after 28 days at 25 °C. The retention rates of crocin (**B**) and quercetin (**C**) of W/O/W emulsions at 25 °C after 28 days of storage. The values are shown as means ± SD of triplicate determinations.

The retention rates of the crocin solution, quercetin solution and W/O/W emulsions are presented in Figure 6B,C, over 28 days at 25 °C. The retention rate of the fresh emulsion was less than 100%, due to the thermal ultrasonic breaking of the emulsions during the assay process, resulting in the partial degradation of crocin and quercetin. In the W/O/W emulsions containing proteins, the retention rates of both crocin (exceeding 64.90%) and quercetin (surpassing 80.29%) were significantly higher than those in the control and free pure solutions. This implied that the W/O/W emulsion can effectively shield bioactives, and the synergistic application of PGPR and proteins further improved their stability. Firstly, the W/O/W emulsions stabilized by the proteins and PGPR exhibited increased viscosity, reducing the flocculation and coalescence among the oil droplets (Figure 6A), consequently averting emulsion rupture and destabilization. Secondly, the combination of proteins and PGPR reduced the agglomeration of the internal water droplets (Figure 6A), which can avoid the formation of reverse micelles induced by the excess emulsifier. Finally, the proteins adsorbed at the interface within the emulsion system exhibited antioxidant properties [47]. In addition, quercetin could protect crocin from degradation due to its antioxidant properties. Compared to WPI, the combination of PGPR and PPI or CPI performed better at protecting crocin (more than 70.04%) and quercetin (more than 84.95%) at the PGPR/protein ratios of 4:1 and 3:2. The W/O/W emulsion droplets, stabilized by PGPR and PPI or CPI, exhibited a relatively uniform dispersion, structural integrity and reduced internal water droplet leakage (Figure 6A). Moreover, the superior interfacial activity and enhanced antioxidant properties of PPI and CPI enabled it to be more effective in slowing the oxidation of the oil than WPI [36]. Consequently, these factors aided in mitigating the degradation of bioactive substances.

### 3.8. In Vitro Digestion

**Figure 7:**
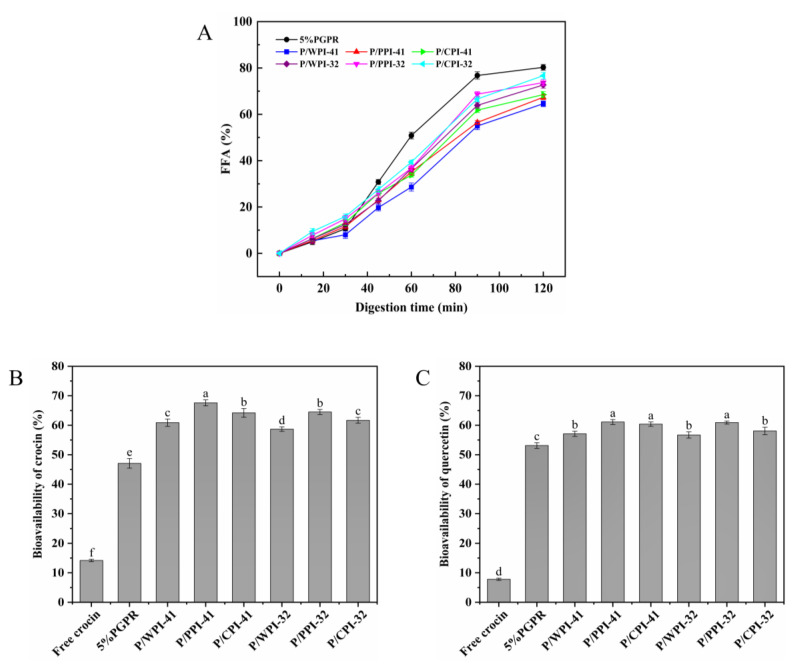
(**A**) Free fatty acids (FFA) release of W/O/W emulsions during simulated gastrointestinal digestion. Bioavailability of crocin (**B**) and quercetin (**C**) after digestion. The values are shown as means ± SD of triplicate determinations. Different lowercase letters represent significant differences (*p* < 0.05) between different W/O/W emulsions.

To evaluate the FFA and bioavailability of the W/O/W emulsions stabilized co-stabilizing by PGPR and proteins, a gastrointestinal digestion simulation was performed. The release of FFA in all the emulsions exhibited an initial slow increase followed by a rapid rise (Figure 7A). This can be ascribed to the WPI–PEC complex in the external aqueous phase, which resulted in an elevation in both the emulsion viscosity and the thickness of the interfacial layer, consequently delaying lipid digestion [48]. Compared to the control, the combination of protein and PGPR resulted in a reduction in FFA release (less than 80%). At PGPR/protein ratios of 4:1 and 3:2, the emulsions containing WPI exhibited a modestly lower FFA release rate (64.57% and 72.66%) after digestion than those of PPI and CPI. The variations in FFA among different protein emulsions appear to correspond to the viscosity and particle size. The emulsions with WPI exhibited larger droplet sizes, resulting in a smaller surface area and reduced contact for the lipase reaction with the oil. Simultaneously, the increased viscosity of emulsions impeded lipase diffusion. Consequently, the emulsions experienced a marginally slower rate of FFA digestion [49].

The bioavailability of crocin and quercetin is presented in Figure 7B,C. The diminished chemical stability of crocin and the limited water solubility of quercetin contributed to their low bioavailability (14.18% and 7.81%, respectively). In the W/O/W emulsion, the bioavailability increased to 47.06% and 53.11% for crocin and quercetin, respectively. The bioactive compounds were encapsulated within a distinctive two-membrane, three-phase structure that shielded them from the gastric environment. The formation of complexes between the proteins and polysaccharides improved the stability of the emulsion during gastric digestion. Upon entering the small intestinal digestion, the mixed micelles resulting from lipid digestion facilitated the solubility and stability of the bioactive compounds [50]. During co-encapsulation, quercetin, which has exceptional antioxidant properties, can also protect crocin. Compared to the control, the bioavailability of co-encapsulated bioactives was further enhanced by incorporating protein into the internal water phase, particularly PPI. At the ratios of 4:1 and 3:2, the bioavailabilities of crocin and quercetin increased by 20.53 and 17.53%, and 7.98 and 7.79%, respectively. This result may be attributed to the propensity of oil droplets to coalesce within more viscous emulsions during gastrointestinal digestion, leading to a decrease in the surface area. This diminished the interaction between the lipase and oil droplets, thereby inhibiting lipid digestion and preventing excessive quercetin loss. Furthermore, the combination of proteins and PGPR formed a more protective physical barrier at the water–oil interface, diminishing the outward migration of crocin. Notably, the bioavailabilities of the bioactives in emulsions containing WPI and PGPR were lower than those of PPI and CPI. This observation may be associated with the outcomes of FFA release. Following intestinal digestion, a greater amount of undigested emulsion with WPI remained, which cannot be converted into micelles [51].

## 4. Conclusions

This study demonstrated that a combination of protein in the internal aqueous phase and PGPR can be used in W/O/W emulsion systems, effectively reducing the usage of PGPR, while improving the stability and encapsulation properties of the emulsions. The results indicated that the encapsulation efficiency of the emulsions decreased in comparison to that of PGPR (control). Nevertheless, the emulsions stabilized by the combination of PPI or CPI with PGPR exhibited superior physical stability. This can be primarily attributed to the outstanding interfacial properties of PPI or CPI absorbed at the water–oil interface. The higher viscosity and enhanced resistance to stress deformation of the emulsions improved the retention of crocin (exceeding 70.04%) and quercetin (exceeding 80.29%) after 28 days of storage. Additionally, this combination bolstered the bioavailability of crocin and quercetin after gastrointestinal digestion. Therefore, proteins can efficiently substitute a portion of PGPR and exhibit potential synergistic effects, significantly enhancing the stability and functionality of W/O/W emulsions. This study contributes to the enrichment of the W/O/W emulsion system and concurrently broadens its commercial application prospect in the food industry. The subsequent research should delve deeper into exploring the synergistic effects of the protein and PGPR combination in W/O/W emulsion systems and investigating stability across diverse environmental conditions.

## Figures and Tables

**Table 1:** Encapsulation efficiency of crocin and quercetin in fresh W/O/W emulsions.

Bioactive Substance	W/O/W Emulsion	Encapsulation Efficiency at Different PGPR/Protein Ratios (%)				
		5:0	4:1	3:2	1:1	2:3
Crocin	P/WPI	86.90 ± 0.67 ^A^	84.39 ± 0.33 ^Ba^	78.12 ± 0.26 ^Ca^	62.79 ± 0.37 ^Da^	56.20 ± 0.76 ^Ea^
	P/PPI	86.90 ± 0.67 ^A^	82.22 ± 0.18 ^Bb^	77.78 ± 0.15 ^Ca^	58.46 ± 0.64 ^Db^	54.63 ± 0.16 ^Eb^
	P/CPI	86.90 ± 0.67 ^A^	81.50 ± 0.14 ^Bc^	71.02 ± 0.34 ^Cb^	56.85 ± 0.96 ^Dc^	52.09 ± 0.55 ^Ec^
Quercetin	P/WPI	94.89 ± 0.38 ^A^	92.80 ± 0.19 ^Ba^	92.44 ± 0.14 ^BCa^	91.78 ± 0.40 ^CDa^	90.21 ± 0.39 ^Da^
	P/PPI	94.89 ± 0.38 ^A^	91.93 ± 0.21 ^Ba^	92.39 ± 0.22 ^Ba^	91.16 ± 0.24 ^Ba^	88.41 ± 1.08 ^Cb^
	P/CPI	94.89 ± 0.38 ^A^	92.10 ± 0.05 ^Ba^	91.23 ± 0.46 ^BCa^	91.14 ± 0.66 ^BCa^	90.23 ± 0.32 ^Ca^

The values are shown as means ± SD of triplicate determinations. ^a–c^ Means with different uppercase letters in the same column are significantly different (*p* < 0.05). ^A–E^ Means with different lowercase letters in the same row are significantly different (*p* < 0.05). The 5:0 PGPR/protein ratio was regarded as the control.

**Table 2:** Particle size, ζ-potential and surface hydrophobicity of WPI, PPI and CPI.

Protein Type	WPI	PPI	CPI
Size (nm)	384.9 ± 3.6 ^a^	221.4 ± 1.6 ^b^	168.6 ± 1.8 ^c^
ζ-potential (mV)	−27.20 ± 0.2 ^c^	−39.10 ± 1.0 ^a^	−37.40 ± 0.7 ^b^
H_0_	105.63 ± 1.30 ^c^	165.10 ± 1.45 ^a^	122.40 ± 1.45 ^b^

The values are shown as means ± SD of triplicate determinations. ^a–c^ Means with different lowercase letters in the same row are significantly different (*p* < 0.05).

## Data Availability

Data is contained within the article or Supplementary Materials.

## Supplementary Materials

The following supporting information can be downloaded at: https://www.mdpi.com/article/10.3390/foods13010131/s1, Table S1: Encapsulation efficiency of W/O/W emulsions at different PGPR concentrations. Table S2: Pseudo-plastic model (v = k·(γ)n) of W/O/W emulsions.
