# Supplementary material for: Effects of the Combination of Protein in the Internal Aqueous Phase and Polyglycerol Polyricinoleate on the Stability of Water-In-Oil-In-Water Emulsions Co-Encapsulating Crocin and Quercetin
Source: Foods. 2023 Dec 29;13(1):131. doi: 10.3390/foods13010131 (PMC10779032; doi:10.3390/foods13010131)
Supplement: Supplementary file 1 [file foods-13-00131-s001.zip › foods-2783136-supplementary.pdf]

Table S1. Encapsulation efficiency of W/O/W emulsions at different PGPR concentrations.

PGPR concentration (%)	Encapsulation efficiency (%)	
	crocin	quercetin
2	58.11 ± 0.35^{d}	91.05 ± 0.31^{b}
2.5	60.05 ± 0.73^{c}	91.42 ± 0.45^{ab}
3	73.42 ± 0.28^{b}	91.63 ± 0.26^{ab}
4	77.76 ± 0.23^{a}	92.28 ± 0.68^{a}

The values are shown as means ± SD of triplicate determinations. a-c Means with different lowercase letters in the same column are significantly different ($P < 0.05$).

Table S2. Pseudo-plastic model ($v=k\cdot(\gamma)^n$) of W/O/W emulsions.

emulsion	k	n	R^2
5%PGPR	1.346	0.666	0.980
WPI-41	4.229	0.618	0.986
PPI-41	3.589	0.615	0.985
CPI-41	3.545	0.628	0.986
WPI-32	3.175	0.634	0.987
PPI-32	2.047	0.639	0.982
CPI-32	1.783	0.660	0.985

v was the viscosity (Pa·s), k was the consistency coefficient ($Pa\cdot s\cdot(s)^n$), γ was the shear rate (1/s) and n was the behavior index (dimensionless).
